# Novel Polyurethane Scaffolds Containing Sucrose Crosslinker for Dental Application

**DOI:** 10.3390/ijms23147904

**Published:** 2022-07-18

**Authors:** Marcell Árpád Kordován, Csaba Hegedűs, Katalin Czifrák, Csilla Lakatos, Ibolya Kálmán-Szabó, Lajos Daróczi, Miklós Zsuga, Sándor Kéki

**Affiliations:** 1 Department of Applied Chemistry, University of Debrecen, Egyetem tér 1, H-4032 Debrecen, Hungary; kordovan.marci@science.unideb.hu (M.Á.K.); czifrak.katalin@science.unideb.hu (K.C.); lakatoscsilla@science.unideb.hu (C.L.); zsuga.miklos@science.unideb.hu (M.Z.); 2 Doctoral School of Chemistry, University of Debrecen, Egyetem tér 1, H-4032 Debrecen, Hungary; 3 Department of Prosthetic Dentistry and Biomaterials, Faculty of Dentistry, University of Debrecen, H-4012 Debrecen, Hungary; hegedus.csaba.prof@dental.unideb.hu (C.H.); szabo.ibolya@med.unideb.hu (I.K.-S.); 4 Department of Solid State Physics, University of Debrecen, Bem tér 18/b, H-4026 Debrecen, Hungary; lajos.daroczi@science.unideb.hu

**Keywords:** poly(ε-caprolactone), polyethylene glycol, polylactic acid diol, sucrose, mechanical testing, biological testing

## Abstract

In this paper, the synthesis, characterization, and properties of crosslinked poly(ε-caprolactone)-based polyurethanes as potential tissue replacement materials are reported. The polyurethane prepolymers were prepared from poly(ε-caprolactone)diol (PCD), polyethylene glycol (PEG)/polylactic acid diol (PLAD), and 1,6-hexamethylene diisocyanate (HDI). In these segmented polyurethanes, the role of PEG/PLAD was to tune the hydrophobic/hydrophilic character of the resulting polymer while sucrose served as a crosslinking agent. PLAD was synthesized by the polycondensation reaction of D,L-lactic acid and investigated by matrix-assisted laser desorption/ionization time-of-flight mass spectrometry (MALDI-TOF MS) and nuclear magnetic resonance spectroscopy (NMR). The crosslinked polyurethane samples (SUPURs) obtained were characterized by attenuated total reflectance Fourier-transform infrared spectroscopy (AT-FT-IR), swelling, and mechanical (uniaxial tensile tests) experiments. The thermo and thermomechanical behavior were studied by differential scanning calorimetry (DSC) and dynamical mechanical analysis (DMA). The viability of dental pulp stem cells was investigated in the case of polyurethanes composed of fully biocompatible elements. In our studies, none of our polymers showed toxicity to stem cells (DPSCs).

## 1. Introduction

The synthesis and characterization of biocompatible and biodegradable polymers are the focus of current materials research [1,2]. These polymers can be found in many fields of our daily life ranging from medicine to 3D printing technology [3,4,5,6]. Among the areas of applications, medicine is of paramount importance as these polymers can serve in drug delivery systems [7] and tissue replacement applications [8,9].

Furthermore, bone replacement e.g., in orthopedics and dentistry [10,11] with appropriate polymers or polymer composites makes it possible to treat various bone diseases and defects that are difficult or impossible to heal. Not surprisingly, there have been huge developments for these materials in recent years, for which biocompatibility is, of course, an indispensable requirement [12]. Nowadays, osteoinductive [13] and osteoconductive [14] scaffold materials are used in dentistry to treat and replace alveolar bone defects.

The constitutional and structural diversity of polyurethanes (PUs) makes them ideal to tailor their properties according to the needs of a given application area [15]. Thus, if PUs are to be used in the field of medicine, the biocompatibility of their building blocks has to be combined with the appropriate strength and mechanical properties of the resulting polymers. Such preferential building blocks can include poly(ε-caprolactone) (PCL), polyethylene glycol (PEG), and polylactic acid (PLA) as polyols acting as soft segments in the PUs, while diisocyanates can be aliphatic, cycloaliphatic or aromatic form the hard segment [16]. However, the use of aromatic diisocyanates in PUs dedicated to medicinal areas is less favored due to the potential formation and migration of aromatic amines to the surrounding tissues [17,18].

Poly(ε-caprolactone)-s (PCLs) and polylactic acid (PLA) as biocompatible synthetic polyesters are common building blocks of biocompatible polymers due to their versatile physical and chemical properties [19,20,21]. Furthermore, PLA has an additional advantage, namely, it can also be produced from natural sources [21]. Polyethylene glycol (PEG) also meets the requirements for biocompatible materials, with low molecular weight variants used as surface modifiers and solvents in various fields [22,23].

The primary role of natural carbohydrates in PUs, in addition to increasing biocompatibility, spans from their applications as fillers to crosslinkers [24]. Carbohydrates, due to the presence of a large number of hydroxyl functional groups, can act as effective tethers to form crosslinked PUs with isocyanates via the formation of urethane bonds [25]. Among other carbohydrates, cellulose nanofibers are often used as a component of dental materials [26,27,28]. Sucrose is so far the most frequently used carbohydrate for this purpose [29,30]. In our previous works, we have successfully employed sucrose alone or in the form of cooligomers formed with 1,6-hexamethylene diisocyanate as a crosslinker/chain extender in the synthesis of PUs [31,32]. In addition to increasing biocompatibility, our research aimed to combine the elasticity of polyurethanes with the reinforcing effect of sucrose.

In this article, we report the synthesis and characterization of novel sucrose-crosslinked PUs films and scaffolds composed of biocompatible elements such as poly(ε-caprolactone)diol (PCD), polyethylene glycol (PEG), polylactic acid diol (PLAD), sucrose, and 1,6-hexamethylene diisocyanate (HDI) to obtain potential tissue replacement materials with appropriate mechanical properties.

## 2. Results and Discussion

### 2.1. Synthesis of Sucrose-Crosslinked Polyurethanes

**Scheme 1:**
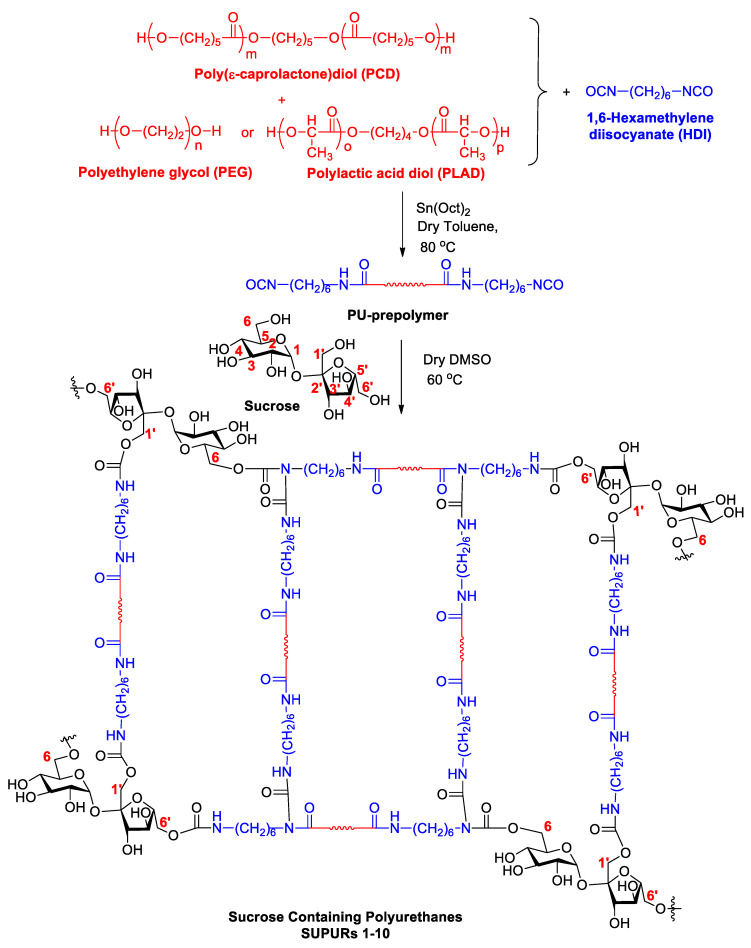
Synthesis pathway for the preparation of SUPURs 1–10.

The goal of this work was the synthesis of polycaprolactone-diol (PCD)-based crosslinked polyurethanes containing polyethylene glycol (PEG)/polylactic acid diol (PLAD) to get biocompatible scaffolds with appropriate mechanical properties. The role of PEG/PLAD was, in addition to influencing the mechanical properties, to tune the hydrophilic/hydrophobic character of the resulting polymers. To investigate the effect of PEG and PLAD on the properties of the polyurethanes formed, their relative amounts were systematically varied. The reaction scheme for the synthesis of crosslinked polyurethanes is depicted in Scheme 1.

**Scheme 2:**
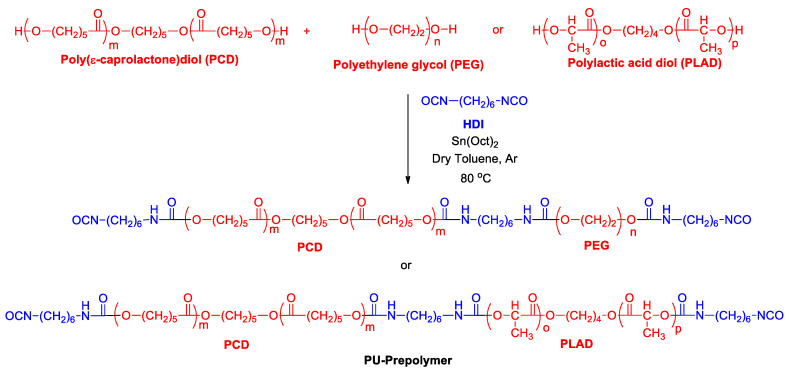
Synthesis of PU-prepolymers.

As seen in Scheme 2, the synthesis of the PU-prepolymers was carried out in toluene solution in the presence of catalyst tin octoate. To form crosslinks, based on one of our previous papers [31], sucrose dissolved in DMSO was added in 0.1 equivalent to PU-prepolymers. As shown in Scheme 1, sucrose participates as netpoints in the formation of the crosslinked structure through its most reactive 6,6′ and 1′ primary hydroxyl groups [33]. The polyurethane prepolymers of different chain lengths can affect flexibility and the crosslink density of the resulting polyurethanes.

The obtained crosslinked polyurethanes (cr-PUs) were then systematically investigated by chemical, mechanical, and thermomechanical methods and tested for dental pulp stem cells (DPSCs).

### 2.2. Infrared Spectroscopy

**Figure 1:**
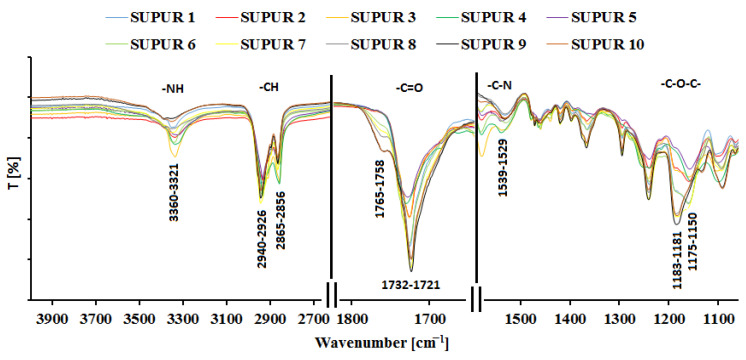
The magnified IR spectra of SUPUR 1–10 samples.

FT-IR spectroscopy was also performed to determine the chemical structure of the prepared SUPUR samples (Figure 1). The stacked IR spectra are compiled in Figure S4. As seen in Figure 1, a low-intensity peak in the absorption range of 3360 to 3321 cm^−1^ referring to the amine–NH stretching vibration of a urethane bond appears in the case of each sample. The presence of the —CH_2_ units in the polymer backbone is revealed by the set of peaks occurring between 2940–2926 cm^−1^ and 2865–2856 cm^−1^. However, the absorption band around 2230 cm^−1^ is missing from the infrared spectrum confirming that no free NCO group is left in the samples. Vibrations of the —C=O group of the caprolactone backbone and urethane bonds can be observed between 1732–1721 cm^−1^. In contrast, in the PLAD-containing samples (SUPURs 6–10), the band belonging to the —C=O group of the PLAD unit appears only as a small shoulder around 1765 cm^−1^. On the other hand, the bands appearing between 1539–1529 cm^−1^ indicate the presence of an amide-II bond. The complex band around 1180 cm^−1^ is due to the presence of —C—O—C— bonds in the interchain ester linkages of polycaprolactone and PLAD segments.

### 2.3. Swelling Experiments

To determine the crosslink density of the resulting polymers, swelling experiments were performed. Our experiments were carried out using toluene as one of the solvents used in the synthesis and the obtained experimental data are summarized in Table 1. From the data in Table 1, it can be surmised that the relative amount of PCD/PEG and PCD/PLAD does not significantly affect the crosslink densities, which are in the order of 10^−3^ mol/cm^3^ for all SUPUR polymers. On the other hand, comparing the results presented in Table 1 it can clearly be concluded that polymers containing PEG reveal the lowest degree of swelling in toluene. This latter finding is in line with the fact that the presence of the hydrophilic PEG in the network increases the hydrophilic character and thus, in turn, decreases the uptake of the nonpolar toluene. Furthermore, PEG-containing samples show the highest gel contents and crosslink densities.

### 2.4. Water Uptake Experiments

**Figure 2:**
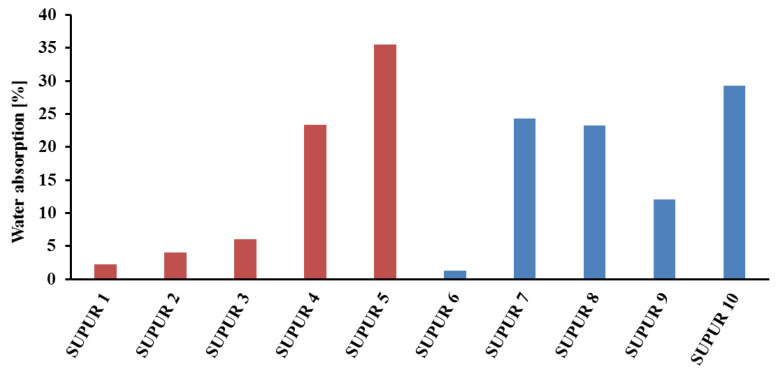
Water absorption by SUPUR 1–10 samples.

Water uptake by a polymer matrix is of important parameter for further biological studies, therefore, we investigated the effect of PEG and PLAD on the swelling properties of the SUPUR samples in water (Figure 2). The data of these swelling experiments are compiled in Table S1.

Figure 2 shows the percentage of water absorption of the SUPUR samples relative to their initial weight. The results on swelling in water show that the water uptake of the SUPUR samples increases steadily with the PEG content. In contrast, this trend is no longer valid when PLAD is present in the samples. However, it can be concluded that water uptake is the highest at the largest PEG contents (SUPUR 5 and SUPUR 10 samples, respectively).

### 2.5. Morphology

**Figure 3:**
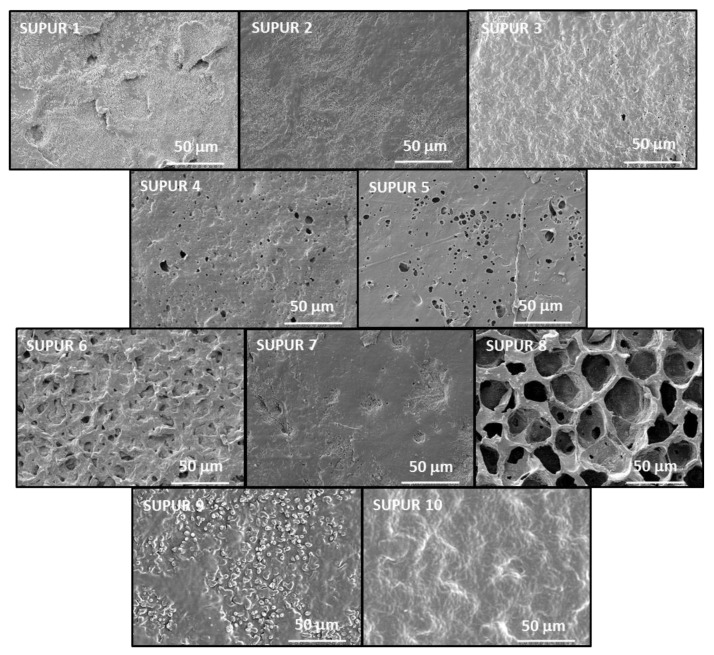
SEM images of SUPUR 1–10 samples.

In order to get deeper insights into the morphology of SUPUR samples, SEM images were taken (Figure 3). Phase separation was observed on some samples, e.g., in the case of SUPUR 1–3, 6, and 8 samples. The higher magnified SEM images (Figure S5) show the fibrous structure, with no regular arrangements suggesting crystallinity. Solvent vapors escaping during the drying of the samples are responsible for the presence of smaller larger cavities in the images of SUPUR 5 and 8 samples.

### 2.6. Mechanical Properties

The data obtained from the uniaxial tensile measurements of SUPURs are presented in Table 2.

As seen from the data in Table 2, except for the SUPUR 5 sample, which has 44 MPa E-moduli with 10% elongation, all samples exhibit low E-moduli and high ultimate strengths. The E-moduli vary between 1.3–6.9 MPa and the elongation at break changes from 830 to 1095% for SUPURs 1–4 and 6–10. In the case of these polymers, the tensile strength and the E-moduli values decrease with the increasing PEG and PLAD content, respectively. This finding is, most likely, due to the plasticizing effect of PEG and PLAD. Comparing the data of the two series (SUPURs 1–5 and 6–10), the tensile strength values of the polymers containing PLAD (SUPURs 6–10) are somewhat lower than those of the SUPUR 1–5 samples. These observations are in line with the crosslink density values (Table 1), where a similar trend can be established.

**Figure 4:**
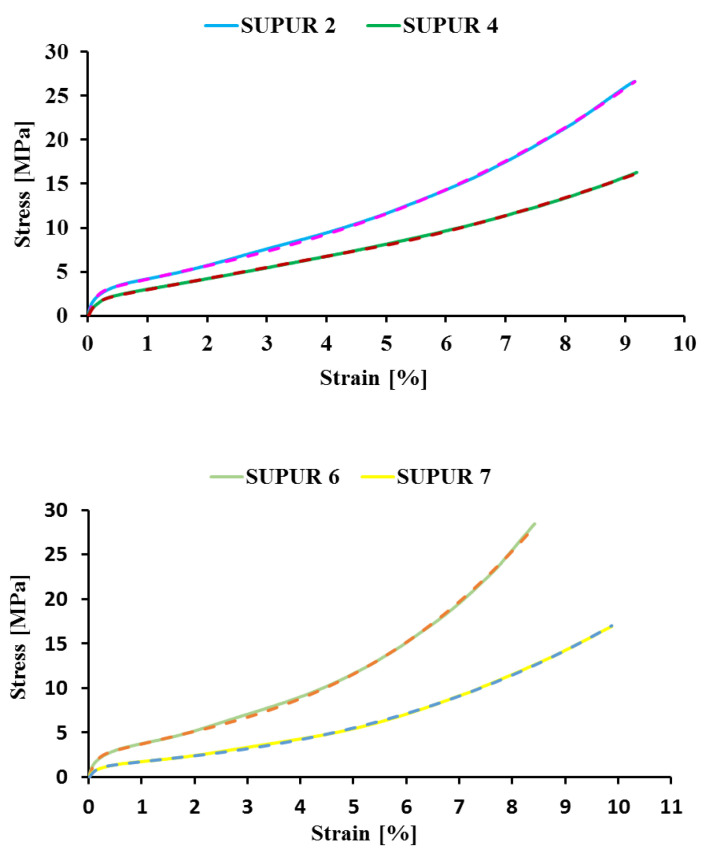
Stress–strain curves of SUPURs 2, 4 and of SUPURs 6, 7. The solid and the dashed lines stand for the experimental and fitted curves according to Equations (1)–(3), respectively.

Furthermore, the uniaxial stress(σ)-strain(ε) curves for SUPUR 1–4 and SUPUR 6–10samples exhibit a strain-hardening effect at higher elongations (ε > 200%) (see Figure 4, Figures S6 and S7). To describe the relationship between σ and ε including the strain-hardening effect, an extended standard linear solid (eSLS) constitutive model is proposed, which has been successfully applied for the characterization of the mechanical properties of various PU systems (Equations (1)–(3)) [31,32,34].

(1) $$d\sigma /d\varepsilon ={\left(\frac{d\varepsilon }{\mathrm{dt}}a_{1}\right)}^{-1}[a_{2}(\varepsilon )\varepsilon +a_{3}(d\varepsilon /\mathrm{dt})-\sigma ]$$

(2) $$a_{2}(\varepsilon )=a_{2}\;\;\;\;\mathrm{if}\;\varepsilon \;\leq \;\varepsilon_{L}$$

and

(3) $$a_{2}(\varepsilon )=a_{2}+\alpha (\varepsilon -\varepsilon_{L}{)}^{\beta }\;\;\;\;\;\mathrm{if}\;\varepsilon \;>\;\varepsilon_{L}$$

where a_1_, a_2_, and a_3_ are the parameters of the SLS model, ε_L_ stands for the onset of strain-hardening, and α and β are the parameters expressing the extent of strain-hardening.

Integrating Equation (1) numerically (after substitution of Equation (2) and with the conditions indicated) and fitting it to the experimental data the parameters of Equations (1)–(3) can be estimated.

As seen in Figure 4 and Figures S6 and S7, the eSLS model describes adequately the experimental stress–strain curves for each SUPUR sample. The fitted β values fall into the range of 1.23–1.64 and 1.45–1.76 for SUPUR samples containing PCD/PEG and PCD/PLAD, respectively, i.e., indicating the more pronounced strain-hardening in the latter case. The values of the fitted parameters are summarized in Table S2.

### 2.7. Thermo- and Thermomechanical Properties of SUPURs 1–10

**Figure 5:**
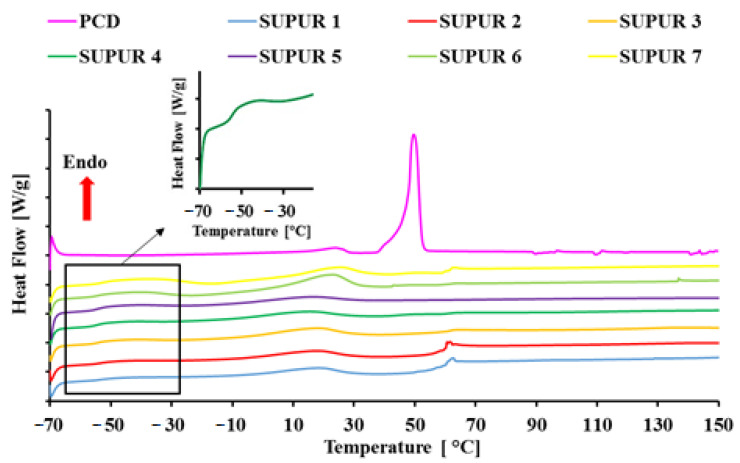
DSC traces for the poly(ε-caprolactone)diol and SUPURs 1–7. The inset is a representation of SUPUR 4. The inflection associated with its T_g_.

The thermal analysis of SUPUR samples was carried out by DSC measurements. The DSC traces are shown in Figure 5 and Figure S9, presenting the thermal transitions (glass transition temperature, T_g_ and melting temperature, T_m_).

As can be seen in Figure 5 and from the data in Table 3, the T_g_ of PCD does not appear because its value is outside the temperature range used during the measurement (T_g_ < −70 °C) [35] However, when the PCD is incorporated into the polyurethane network, glass transition to the rubbery state (T_g_) are observed in the temperature range of −37.6–−54.1 °C. The explanation for this finding is that the chain flexibility decreases upon crosslinking. In contrast, also due to the effect of crosslinks, the melting point of the SUPUR samples decreases with respect to that of the pure PCD. On the other hand, it can also be established that the T_g_ increases with the increasing PLAD content (SUPUR 6–10 samples). Furthermore, the presence of an amorphous state in these SUPUR samples is the most characteristic and no T_m_ could be detected for SUPUR 8–10 samples.

**Figure 6:**
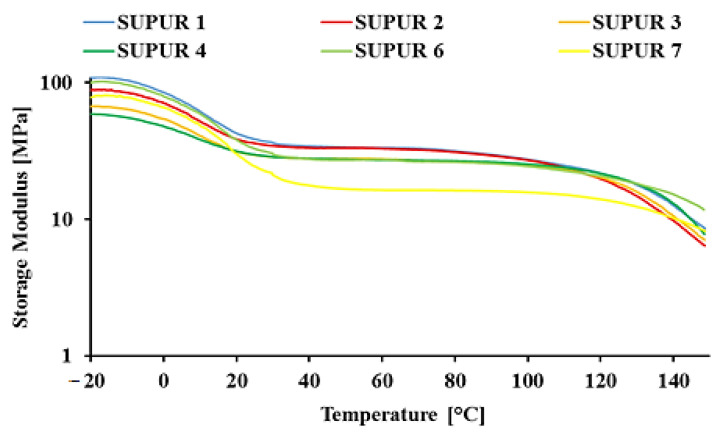
Variation of the storage modulus values (E’) with the temperature for SUPUR 1–4 and SUPUR 6, 7 samples.

The thermomechanical properties of SUPUR samples were evaluated using DMA. The storage modulus versus temperature curves are presented in Figure 6 and Figure S10.

As it turns out from Figure 6, on the storage modulus versus temperature curves for the SUPUR 1–4 and SUPUR 6–9 samples, a well-developed plateau appeared in the temperature range of 35 °C–100 °C that is preceded by a steep decrease in the storage modulus, attributable to the melting of the PCD segment, in the range of 15 °C–30 °C. The presence of the plateau also confirms the formation of the crosslinked structure from which the crosslink density at a given temperature can be calculated using Equation (4) (Table 4).

(4) $$\nu_{e}=\frac{E^{\prime }}{3\mathrm{RT}}$$

where E′, R, and T are the storage modulus, the universal gas constant, and the temperature at the onset of the rubbery state, respectively.

The crosslink densities given in Table 4 are in the order of 10^−3^ mol/cm^3^ consistently with the values determined by swelling experiments. Furthermore, the crosslink density of PLAD-containing polymers is lower than that of PEG samples, in line with the results of the experiments.

### 2.8. Porosity Measurement of Scaffolds

The scaffolds prepared according to the method detailed in the experimental section were examined using SEM. Based on the SEM image parameters characterizing such as the pore sizes, ellipse major and minor as well as the Feret diameters of the scaffolds were determined. ImageJ software was used to determine the pore size of the scaffolds [36,37].

**Figure 7:**
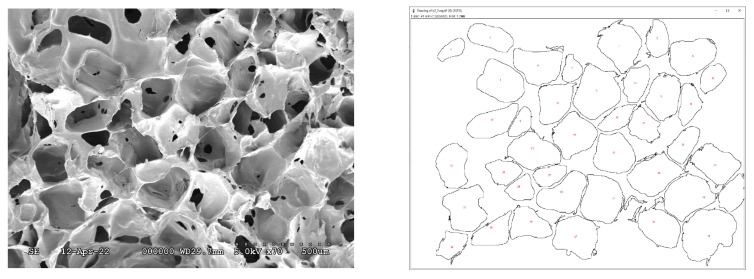
The SUPUR 2 scaffold’s SEM image (**left**) and the outlines of the pores examined in the SUPUR 2 sample (**right**).

The results of the analysis are compiled in Table 5 and Table 6. As an example, the SEM picture of the scaffold of the SUPUR 2 sample together with the outline of the pores is shown in Figure 7.

It can be surmised from the data in Table 5 and Table 6 that no significant change in the corresponding pore parameters with the composition of the samples can be observed. This finding confirms that these parameters are primarily determined by the applied salt particles and that the polymer matrices ranging from SUPUR 1 to SUPUR 7 samples are mechanically capable of adopting the sizes of the leaching particles. Moreover, as it also turned out from Table 6, SUPUR 8, 9, and 10 samples are not enabled to form well-developed scaffolds. The SEM images of SUPUR scaffolds 1, 3–5, and 6–10 are presented in Figure S9.

### 2.9. Cell Viability Assay

**Figure 8:**
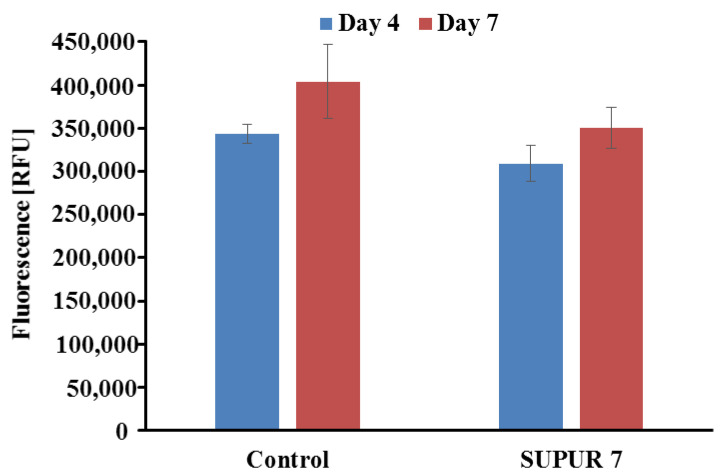
Cell viability assay of glass surface (control) and SUPUR 7 scaffold. (Two-sample T-test: *p* ≥ 0.05).

To confirm the biocompatibility of the samples, as an example, SUPUR 7 samples were tested for dental pulp stem cells (DPSCs). The cell viabilities obtained on the different surfaces are shown in Figure 8.

**Figure 9:**
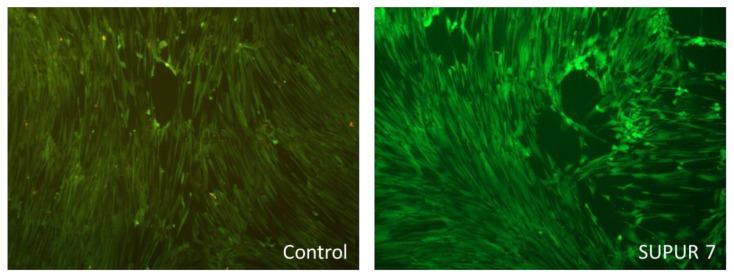
Live/dead assay of glass surface control and SUPUR 7 by fluorescent microscopy.

As a control, the cells were seeded onto a glass surface, and the scaffold material surface was used to detect cytocompatibility. After 7 days the assays showed the sample did not cause a significant change in cell viability compared to the control (*p* ≤ 0.05), using a two-sample T-test (number of control: 4, number of test item: 12), cell viability of SUPUR 7 sample was compared to the glass surface control. These results revealed that the scaffold has good biocompatibility for long-term use. A live/dead cytotoxic assay of control and SUPUR 7 was conducted on day 7 (Figure 9).

Based on fluorescein staining and analysis of the nuclei, Figure 9 shows that DPSCs grew steadily and proliferated on the scaffolds over time, and most of the cells remained viable at all-time examined. In addition, the fraction of dead cells appeared to be higher in the control sample.

## 3. Materials and Methods

### 3.1. Materials

For the polyurethane synthesis poly(ε-caprolactone)diol (PCD, M_n_ = 2000 g/mol), polyethylene glycol (PEG, M_n_ = 600 g/mol), 1,6-hexamethylene diisocyanate (HDI, reagent grade, 98%), tin(II) 2-ethylhexanoate (Sn(Oct)_2_), M_n_ = 405.12 g/mol, 92.5–100%), and 1,4-butanediol (BDO) Reagent Plus^®^ (99%) M = 90.12 g/mol were purchased from Sigma-Aldrich (Darmstadt, Germany) and D,L-lactic acid (LA, 88% assay, from Spektrum 3D Ltd. Debrecen, Hungary) was used. D(+)-sucrose (puriss, Ph. Eur. 6.) from Reanal (Budapest, Hungary) was powdered and dried in a vacuum oven at 40 °C overnight before use as a crosslinker. Toluene (analytical grade) and dimethyl-sulfoxide (99.9%, DMSO, stored in a molecular sieve) from Sigma-Aldrich (Darmstadt, Germany) were used as solvents without any purification. Sodium chloride (a.r.) from Molar Chemicals Ltd. (Halásztelek, Hungary) was used to form the scaffold. Reverse osmosis filtered water (RO) was used for the dissolution of salt. The RO was produced with an HFRO1.0 device manufactured by Hidrofilt Ltd. (Nagykanizsa, Hungary).

### 3.2. Synthesis

#### 3.2.1. Synthesis of Polylactic Acid Diol (PLAD)

PLAD was synthesized as described elsewhere [38]. D,L-lactic acid (LA) (50 g, 88%) was dehydrated at 70 °C and 20 Hgmm for 2 h to obtain 39.2 g (435 mmol) viscous yellowish material. PLA-diol (PLAD) was synthesized by the reaction of the previously obtained dehydrated lactic acid (LA) with 1,4-butanediol (BDO) (5 mole%) in the presence of tin(II) 2-ethylhexanoate Sn(Oct)_2_ (0.1 mole%) catalyst. The viscous mixture was heated at 160 °C and 40 Hgmm for 7 h. Then the reaction mixture was cooled down to room temperature to yield 32.2 g of yellowish thick oil with a yield of 96%, which was examined by NMR spectroscopy [39] (M_n_ = 824 g/mol, ^1^H-NMR and ^13^C-NMR spectra are presented in the supporting information in Figures S1 and S2), by GPC (M_n_ = 938 g/mol, PDI = 1.36) and by MALDI-TOF MS (M_n_ = 1000 g/mol, Figure S3) to determine the number average molecular weight (M_n_) and the polydispersity index (PDI = M_w_/M_n_). The obtained PLAD was used for further reactions without any purification.

#### 3.2.2. Synthesis of PU-Prepolymer for SUPUR 1

The synthesis of PU-prepolymer was carried out in dry toluene under an argon atmosphere (Scheme 2). In a 100 mL round-bottom, three-necked flask (previously dried at 140 °C) equipped with a mechanical stirrer, argon inlet, and reflux condenser with an argon outlet. The flask was charged with 9.00 g (4.50 mmol) of PCD (2000 g/mol), 0.30 g (0.50 mmol) of PEG (600 g/mol), and 50.0 mL of dry toluene under continuous argon flow at 80 °C. After complete dissolution PCD and PEG 1.65 mL, 1.68 g (10.0 mmol) HDI, and 0.02 g (0.05 mmol) of tin(II) 2-ethylhexanoate as catalyst was introduced. The homogeneous mixture was stirred at 80 °C for four hours. The same procedure was applied for the synthesis of PCD-PLAD prepolymers.

#### 3.2.3. Crosslinking by Sucrose

A pre-calculated amount of dried sucrose (0.17 g (0.50 mmol)) was dissolved in anhydrous DMSO of 3.0 mL at 60 °C. After the complete dissolution of sucrose, the DMSO solution was added in one portion to the PU-prepolymer solution during vigorous stirring and the temperature was kept at 60 °C for two hours. Then the resulting viscous mixture was poured into a Teflon pan and cured at 60 °C for one night, and dried at room temperature until constant weight to give 6.56 g (60%) a clear elastic polymer sheet.

The compositions of the SUPUR samples are shown in Table 7 and Table 8.

#### 3.2.4. Synthesis of SUPUR Scaffolds by Salt Leaching

The preparation of the scaffold up to the crosslinking step was the same as previously described [40]. Before pouring into the pan, ground and graded NaCl with a diameter of 200 to 250 µm, and a 7-fold weight of the polymer was added and mixed until it was homogeneous. The reaction mixture was placed in an oven at 60 °C for one day. This condition resulted in a sheet of about 5 mm thick measured by a caliper. The sample was cut into smaller pieces and soaked in heat sterilized RO water to wash out the salt. The water was refreshed every day. Dissolution of the salt was performed until the conductivity of the sterilized water had not changed. The sample was then stored in the oven at 40 °C overnight. The resulting scaffolds were flexible, open-cell sponge-like materials.

### 3.3. Characterization

The number- (M_n_) and the weight-average molecular weight (M_w_) and the polydispersity (M_w_/M_n_) of PLAD prepared were determined by size-exclusion chromatography (SEC). The chromatograms were recorded in tetrahydrofuran (THF) at a flow rate of 0.5 mL/min with a Waters chromatograph equipped with four gel columns (4.6 × 300 mm, 5 µm Styragel: HR 0.5, 1, 2 and 4), Waters Alliance 2695 HPLC pump, and with a Waters 2414 refractive index detector. The chromatograph was calibrated using polystyrene standards. The PLAD was dissolved in THF at a concentration of 5 mg/mL and filtrated by a syringe filter (porosity: 0.45 μm).

The ^1^H- and ^13^C-NMR spectra were recorded from PLAD with a Bruker AM360 (360/90 MHz for ^1^H/^13^C) spectrometer in deuterated chloroform. Chemical shifts were referenced to the ^1^H signal of Me_4_Si.

Further investigation of the structure of PLAD was executed by matrix-assisted laser desorption/ionization time-of-flight mass spectrometry (MALDI-TOF MS). The measurements were carried out with a Bruker Autoflex Speed mass spectrometer equipped with a time-of-flight/time-of-flight (TOF/TOF) mass analyzer. In each case, 19 kV acceleration voltage was employed and the ions were detected in positive ion mode. Appreciable resolution and mass accuracy were obtained in the reflectron mode. A total of 21 kV and 9.55 kV were applied as the reflector voltages 1 and 2, respectively. A solid-phase laser (355 nm, ≥100 μJ/pulse) operating at 500 Hz was applied to produce laser desorption and, 5000 shots were summed. The MALDI-TOF MS spectra were externally calibrated with polyethylene glycol standard (M_n_ = 1540 g/mol). Samples for MALDI-TOF MS measurements were prepared with 2,5-dihydroxy benzoic acid (DHB) matrix dissolved in THF at a concentration of 20 mg/mL. The samples and sodium trifluoroacetate used as an ionizing agent were also dissolved in THF at a concentration of 10 mg/mL and 5 mg/mL, respectively. The mixing ratio was 10/2/1 (matrix/sample/cationizing agent). A volume of 0.25 μL of the solution was deposited onto a metal sample plate and allowed to air dry.

Attenuated total reflectance (ATR) Fourier-transform infrared (FT-IR) spectra were recorded on a Perkin Elmer Instruments Spectrum Two FT-IR spectrometer equipped with a diamond Universal ATR Sampling Accessory. The average film thickness of the specimens varied in the range of 0.6–1.0 mm. Four scans were gathered for each sample. The IR spectra were then assessed using the Spectrum ES 5.0 program.

Swelling experiments were carried out to determine the crosslink density. The samples (dimension: 10 mm × 10 mm × ~0.3 mm) were swollen in toluene (10 mL) at 22 °C (295 K) in a closed bottle for 24 h according to the procedure presented in ref [41]. The degree of swelling (Q) and the gel content (G) was calculated by Equations (5) and (6) [41,42]:

(5) $$Q=1+\;\frac{\rho_{s}}{\rho_{p}}\left(\frac{m_{2}}{m_{3}}-1\right)$$

(6) $$G\left(\%\right)=\frac{m_{3}}{m_{1}}\cdot 100$$

where ρ_s_ and ρ_p_ are the densities of the solvent (toluene, ρ: 0.8669 g/cm^3^) and SUPUR polymers. The masses m_1_, m_2,_ and m_3_ are the initial weight, swollen weight, and constant weight for the SUPUR polymers, respectively.

The crosslink density of the SUPUR samples (ν_e_) was calculated by means of the Flory–Rehner equation (Equation (7)) [43]:

(7) $$\nu_{e}=\frac{-\left[\ln\left(1-v_{1}\right){+v}_{1}+\chi \;\cdot {\;v}_{1}{}^{2}\right]}{V_{\mathrm{ms}}\cdot \;\left(v_{1}{}^{1/3}-\frac{v_{1}}{2}\right)}$$

where v_1_ is the volume fraction of the polymer and V_ms_ is the molar volume of the solvent.

The solvent–polymer interaction parameter (χ) was determined by Equation (8),

(8) $$\chi =\frac{{\left(\delta_{2}-\delta_{1}\right)}^{2}\cdot {\;V}_{\mathrm{ms}}}{R\;\cdot T}$$

where δ_1_ and δ_2_ are the solubility parameter of the solvent and polymer, respectively; R is the universal gas constant and T is the absolute temperature. The Hildebrand solubility parameters of toluene and PU polymers were δ_1_ = 18.2 and δ_2_ = 20.5 (MPa)^1/2^, respectively [44]. The molar volume of toluene (V_ms_) and interaction parameter (χ) at 302 K was calculated to be 1.06 × 10^−4^ m^3^/mol and 0.223, respectively.

In parallel with the swelling experiment, water uptake was examined on 10 mm × 10 mm ~0.3 mm size samples. One sample per type was swelled in 50 mL of water for 48 h. The mass of the samples was measured before (m_1_) and after (m_2_) swelling.

Surface morphology was investigated by scanning electron microscopy (SEM). SEM images were taken from the surface of the selected specimens by a Hitachi S-4800 microscope (Tokyo, Japan) equipped with a Bruker energy-dispersive X-ray spectrometer. For microscopic examinations, 1 × 1 cm samples with an average thickness of 0.5 mm were excised. The specimens’ surface was covered with a 30 nm conductive gold layer. The SEM observations were performed at 15 kV accelerating voltage in the secondary electron mode.

For mechanical investigation of SUPUR samples, uniaxial tensile tests were carried out according to the EN ISO 527-1 standard. Computer-controlled Instron 3366 (Instron, Norwood, MA, USA) type tensile testing machine, equipped with a 10 kN load cell, was used. At least 5 dumbbell specimens were cut (clamped length: 60 mm) based on ASTM D882-12 standard and tensile loaded at a crosshead speed of 50 mm/min.

The thermal properties of the synthesized PUs were monitored by differential scanning calorimetry (DSC). DSC measurements were performed with a DSC Q2000 power compensation equipment operating at a 10 °C/min heating rate. Nitrogen flushing was employed as a protective atmosphere. During DSC measurements heat/cool/heat cycles were carried out. Heating cycle: the temperature was raised from −70 °C–220 °C by a 10 °C/min heating rate. Cooling cycle: the sample cooled down from 220 °C–−70 °C using the same heating rate.

Dynamic mechanical analysis (DMA) testing of the PU samples was carried out with METRAVIB DMA 25 Instruments. DMA traces were recorded in tension mode (dimension of the specimens: length: 30 mm, clamped length: 18 mm, width: 15 mm, and thickness: ca. 0.5 mm) A dynamic displacement of 0.1 mm was used at frequencies of 10 Hz. The temperature varied between −30 °C and 150 °C with a heating rate of 3 °C/min.

The cell viability on the surfaces of SUPURs was examined by Alamar Blue assay^®^ (Thermo Scientific, Waltham, MA, USA) for dental pulp stem cells (DPSCs). A total of 4 × 10^4^ cells were seeded to the surface of the investigated materials, then cultured for 14 days in DMEM F12 (Gibco, Waltham, MA, USA) supplemented with 10% (*v*/*v*) fetal bovine serum (Gibco), 1% Glutamax (Gibco), and 1% Antibiotic-Antimycotic (Gibco). The culture medium was refreshed 3 times a week. Finally, the Alamar Blue assay was performed according to the manufacturer’s instructions, and the fluorescence of samples (100 µL) was measured using a microplate reader (HIDEX Sense Turku, Finland) at 544 nm excitation/595 nm emission.

Viability staining: DPSCs cells (105/well) were seeded on UV-light-treated (30 min) fiber-covered glass coverslips and incubated for 7 days. Glass surfaces were used as controls. After the incubation period, the cells were co-stained with fluorescein diacetate (FDA) and propidium iodide (PI) (both from Sigma-Aldrich) for 5 min at room temperature. Pictures were taken using a Zeiss AxioVert A1 inverted fluorescence microscope (Carl Zeiss Microscopy GmbH, Jena, Germany). The details about the dental pulp stem cells used in this study can be found in the Supplementary Information.

Statistical analysis of the results was performed using the Microsoft Excel Data Analysis ToolPak.

Cellular test authorized by the Hungarian National Public Health and Medical Officer Service (NPHMOS). Approval code: IF-14135-8/2016.

## 4. Conclusions

Novel polyurethanes composed of biocompatible constituents for potential dental applications have been synthesized. Thus, PEG/PLAD-containing polycaprolactone-based PU-prepolymers were prepared. The PLAD incorporated in the prepolymer was prepared by polymerization starting from lactic acid and 1,4-butanediol. Sucrose was used as a crosslinking agent to form a network that was pointed out to play an important role in determining the mechanical properties of the polyurethanes. The crosslinked SUPUR samples were then investigated by ATR FT-IR, swelling test, mechanical, thermo, and thermomechanical methods, as well as a cell viability assay test. It was confirmed that high crosslinking density associated with a high degree of elasticity resulting in the appearance of a well-developed rubbery plateau on the storage modulus versus temperature curve was achieved. From the SUPUR 1–7 samples, open cell scaffolds were produced using the salt leaching method and no toxicity was observed for the scaffolds as demonstrated by the cell viability test for the dental pulp stem cells in the case of the SUPUR 7 sample. The results are encouraging for the possible use of these polyurethanes as tissue scaffolds in the future.

## Figures and Tables

**Table 1:** Density, degree of swelling (Q), gel content (G), volume fraction of the polymer (v_1_), and crosslink density (ν_e_) at 295 K of SUPURs 1–10 in toluene.

Sample Name	Density (g/cm^3^)	Q	G (%)	v_1_	Crosslink Density (ν_e_) (mol/cm^3^)
SUPUR 1	1.23	1.72	97.7	0.420	2.2 × 10^−3^
SUPUR 2	1.16	1.74	95.8	0.452	2.6 × 10^−3^
SUPUR 3	1.15	1.80	95.1	0.442	2.5 × 10^−3^
SUPUR 4	1.09	1.88	92.3	0.459	2.7 × 10^−3^
SUPUR 5	0.97	2.36	90.6	0.411	2.1 × 10^−3^
SUPUR 6	1.14	1.88	96.9	0.412	2.1 × 10^−3^
SUPUR 7	1.18	2.13	85.5	0.386	1.8 × 10^−3^
SUPUR 8	1.16	2.16	88.4	0.373	1.7 × 10^−3^
SUPUR 9	1.18	2.25	86.4	0.357	1.5 × 10^−3^
SUPUR 10	1.18	2.27	74.8	0.416	2.1 × 10^−3^

**Table 2:** Uniaxial tensile mechanical properties of SUPURs 1–10. Designations: E is the elastic modulus (Young’s modulus), ε_R_ is the ultimate elongation, and σ_R_ is the stress at break.

Sample Name	E (MPa)	ε_R_ (%)	σ_R_ (MPa)
SUPUR 1	3.8 ± 0.1	919 ± 11	23 ± 0.5
SUPUR 2	4.6 ± 0.6	988 ± 63	26 ± 2.9
SUPUR 3	4.5 ± 0.3	921 ± 37	22 ± 2.0
SUPUR 4	2.6 ± 0.5	905 ± 16	16 ± 1.8
SUPUR 5	44 ± 5.5	10 ± 6	5 ± 0.5
SUPUR 6	6.9 ± 0.1	829 ± 11	27 ± 0.8
SUPUR 7	3.1 ± 0.1	1020 ± 26	17 ± 0.5
SUPUR 8	2.9 ± 0.1	1047 ± 18	17 ± 0.7
SUPUR 9	1.7 ± 0.2	935 ± 46	10 ± 1.3
SUPUR 10	1.3 ± 0.1	1095 ± 48	7 ± 1.5

**Table 3:** Glass transition temperature (T_g_) and melting temperature (T_m_) derived from onset temperature obtained from DSC measurements for PCD and SUPURs 1–10 by the analysis of the first heating cycle.

Sample Name	T_g_ (°C)	T_m_ (°C)
PCD(2)	-	47
SUPUR 1	−53.8	18.1
SUPUR 2	−54.1	17.3
SUPUR 3	−54.1	17.3
SUPUR 4	−55.1	14.4
SUPUR 5	−54.6	15.1
SUPUR 6	−54.3	22.7
SUPUR 7	−52.7	25.0
SUPUR 8	−48.5	-
SUPUR 9	−41.3	-
SUPUR 10	−37.6	-

**Table 4:** The crosslink densities from the storage modulus curves of SUPURs 1–4 and 6–9.

Sample Name	ν_d_ (mol/cm^3^)
SUPUR 1	3.9 × 10^−3^
SUPUR 2	3.8 × 10^−3^
SUPUR 3	3.3 × 10^−3^
SUPUR 4	3.2 × 10^−3^
SUPUR 6	3.1 × 10^−3^
SUPUR 7	1.9 × 10^−3^
SUPUR 8	2.4 × 10^−3^
SUPUR 9	1.9 × 10^−3^

**Table 5:** Pore sizing results of PEG-600 modified scaffolds.

Parameters	SUPUR 1	SUPUR 2	SUPUR 3	SUPUR 4	SUPUR 5
Count	93	77	73	46	72
Major diameter (µm)	236 ± 64	250 ± 67	243 ± 57	245 ± 100	246 ± 78
Minor diameter (µm)	146 ± 44	169 ± 47	164 ± 50	154 ± 38	139 ± 46
Ellipse angle	98.7 ± 52	83.2 ± 52	101 ± 51	82.8 ± 54	85.0 ± 55
Feret max (µm)	262 ± 75	274 ± 78	273 ± 72	284 ± 117	307 ± 130
Feret min (µm)	160 ± 49	182 ± 50	182 ± 57	181 ± 55	177 ± 74
Feret angle	107 ± 49	74.9 ± 54	106 ± 47	66.8 ± 49	83.0 ± 56

**Table 6:** Pore sizing results of PLAD-1000 modified scaffolds.

Parameters	SUPUR 6	SUPUR 7	SUPUR 8	SUPUR 9	SUPUR 10
Count	70	86	-	-	-
Major diameter (µm)	295 ± 86	259 ± 72	-	-	-
Minor diameter (µm)	195 ± 64	169 ± 45	-	-	-
Ellipse angle	86 ± 45	73.7 ± 39	-	-	-
Feret max (µm)	314 ± 93	287 ± 80	-	-	-
Feret min (µm)	207 ± 68	188 ± 51	-	-	-
Feret angle	89 ± 45	75.9 ± 38	-	-	-

**Table 7:** The compositions of the SUPUR 1–5 samples.

Sample Name	Prepolymer	PCD-PEG/Sucrose Mole Ratio
	PCD/PEG/HDI Mole Ratio	
SUPUR 1	0.9/0.1/2.0	10/1
SUPUR 2	0.8/0.2/2.0	10/1
SUPUR 3	0.7/0.3/2.0	10/1
SUPUR 4	0.6/0.4/2.0	10/1
SUPUR 5	0.5/0.5/2.0	10/1

**Table 8:** The compositions of the SUPUR 6–10 samples.

Sample Name	Prepolymer	PCD-PLAD/Sucrose Mole Ratio
	PCD/PLAD/HDI Mole Ratio	
SUPUR 6	0.9/0.1/2.0	10/1
SUPUR 7	0.8/0.2/2.0	10/1
SUPUR 8	0.7/0.3/2.0	10/1
SUPUR 9	0.6/0.4/2.0	10/1
SUPUR 10	0.5/0.5/2.0	10/1

## Data Availability

Data are available from the corresponding author upon request.

## Supplementary Materials

The following supporting information can be downloaded at: https://www.mdpi.com/article/10.3390/ijms23147904/s1. References are cited in [45,46].
