# Supplementary material for: Novel Polyurethane Scaffolds Containing Sucrose Crosslinker for Dental Application
Source: Int J Mol Sci. 2022 Jul 18;23(14):7904. doi: 10.3390/ijms23147904 (PMC9319899; doi:10.3390/ijms23147904)
Supplement: Supplementary file 1 [file ijms-23-07904-s001.zip › ijms-1814888-supplementary.pdf]

Supplementary Information for

Novel Polyurethane Scaffolds Containing Sucrose Crosslinker for Dental Application

Marcell Árpád Kordován [1,2], Csaba Hegedűs [3], Katalin Czifrák [1], Csilla Lakatos [1]; Ibolya Kálmán-Szabó[3]; Lajos Daróczi[4], Miklós Zsuga[1] and Sándor Kéki[1,*]

[1] Department of Applied Chemistry, University of Debrecen, Egyetem tér 1, H-4032 Debrecen, Hungary; kordovan.marci@science.unideb.hu (M.Á.K.); czifrak.katalin@science.unideb.hu (K.C.); lakatoscsilla@science.unideb.hu (C.L.); zsuga.miklos@science.unideb.hu (M.Z.)

[2] Doctoral School of Chemistry, University of Debrecen, Egyetem tér 1, H-4032 Debrecen, Hungary

[3] Department of Prosthetic Dentistry and Biomaterials, Faculty of Dentistry, University of Debrecen, 4012 Debrecen, Hungary; hegedus.csaba.prof@dental.unideb.hu (C.H.); szabo.ibolya@med.unideb.hu (I. K.-S.)

[4] Department of Solid State Physics, University of Debrecen, Bem tér 18/b, H-4026 Debrecen, Hungary; daroczi.lajos@science.unideb.hu (L.D.)

* Correspondence: keki.sandor@science.unideb.hu; Tel.: +36-52-512-900 (ext. 22455)

Table of contents

Figure S1. The ^{1}H-NMR spectrum of PLAD..3

Figure S2. ^{13}C-NMR spectrum of PLAD………………………………………..….........4

Figure S3. MALDI-TOF MS spectrum of PLAD………………………………………...4

Figure S4. Stacked IR spectra of SUPURs 1 - 10..……………………………………….5

Table S1. The masses m_1 and m_2 are the initial weight, and swollen weight in water for the SUPUR polymers, respectively……………………..……………………………….………….5

Figure S5. Magnified SEM images of SUPURs 1 - 3 and 6, 8…………………….……....6

Figure S6. The stress-strain curves of SUPURs 1 and 3. The solid lines reperesented the experimental data, while dashed lines stand for fitted curves………………………………6

Figure S7. The stress-strain curves of SUPURs 8 - 10. The solid lines reperesented the experimental data, while dashed lines stand for fitted curves………………………………7

Table S2. The fitted parameters of Equations 5 - 7 for samples SUPUR 1 - 4 and 6 - 10.…7

Figure S8. The stress-strain curves of SUPUR 5…………………………………………...8

Figure S9. DSC curves of SUPUR 8 - 10…………………………………………………..8

Figure S10. Storage modulus curves of SUPUR 8 - 10……………………………………9

Figure S11. SEM images of SUPUR 1 - 10 scaffolds…………………………..……….10

Details about the dental pulp stem cells used in this study………………………….……..11

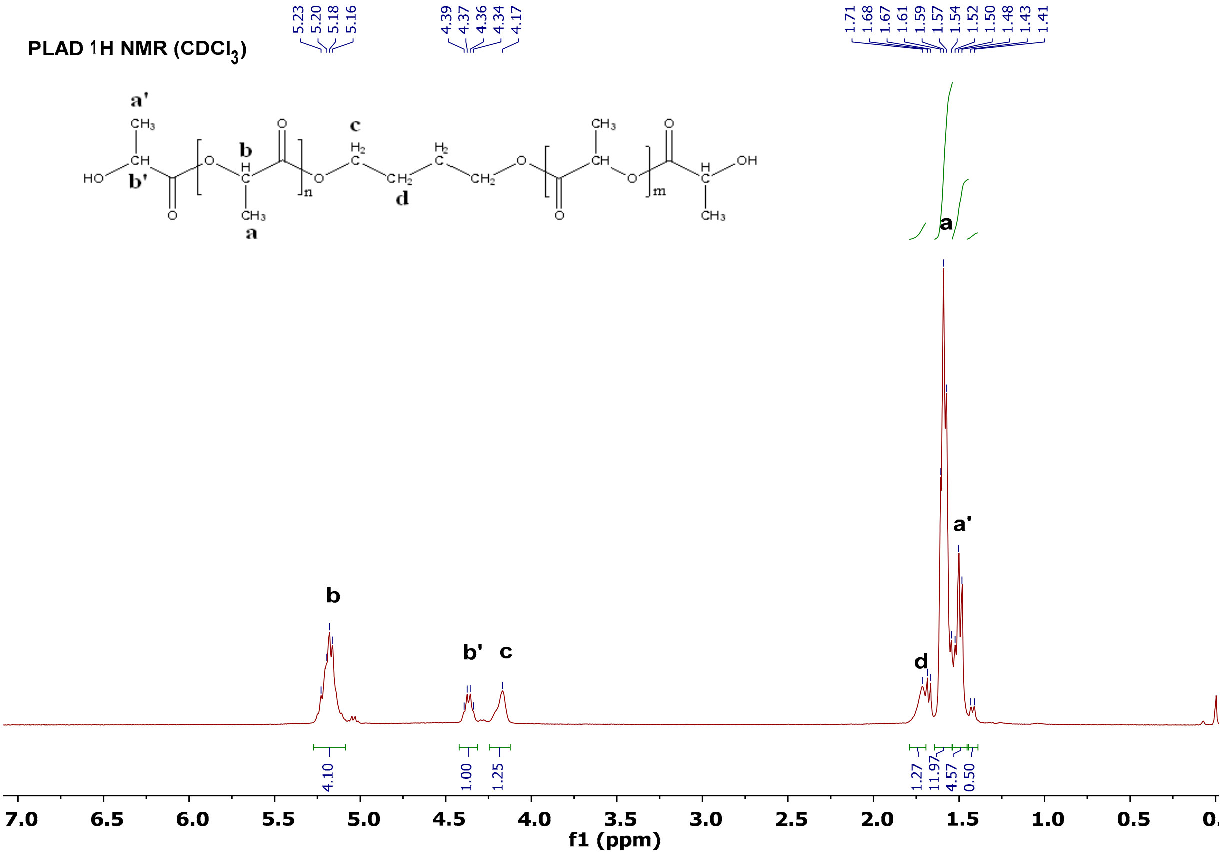

Figure S1. The ^{1}H-NMR spectrum of PLAD.

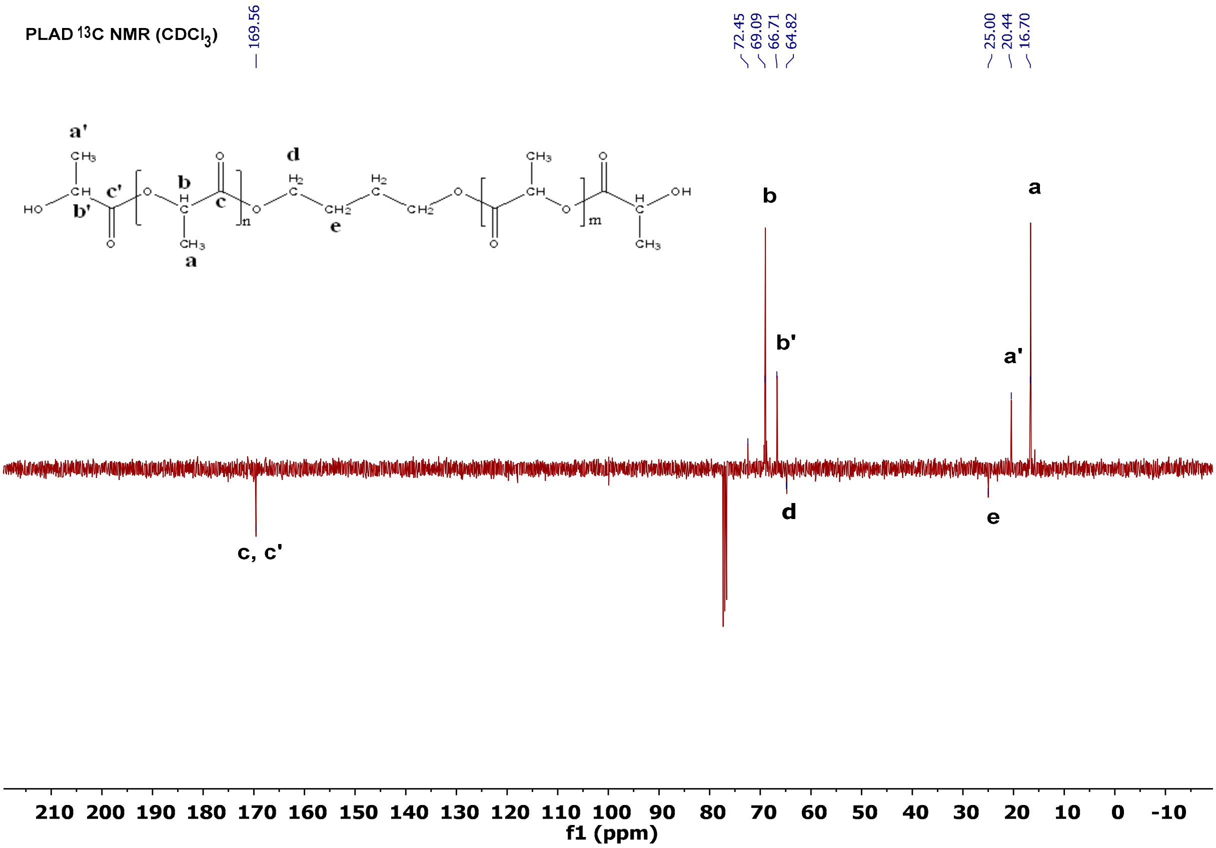

Figure S2. ^{13}C-NMR spectrum of PLAD.

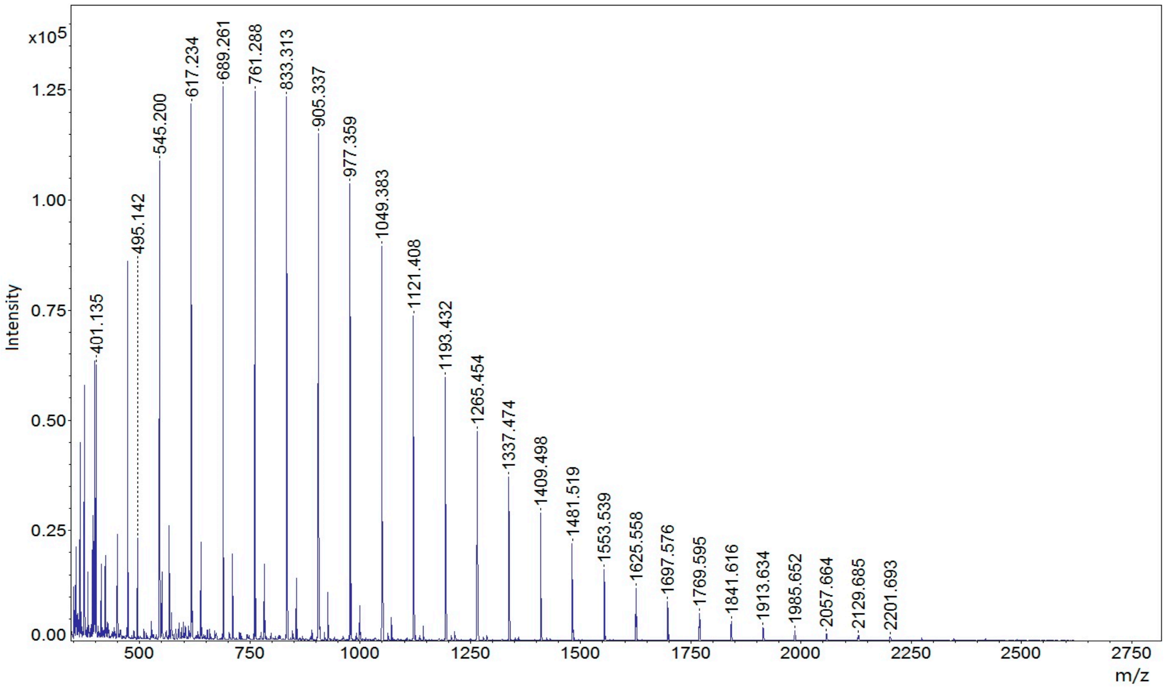

Figure S3. MALDI-TOF MS spectrum of PLAD.
(The lower intensity series belongs to the PLA homopolymer, the ratio of PLAD : PLA homopolymer / 85 : 15)

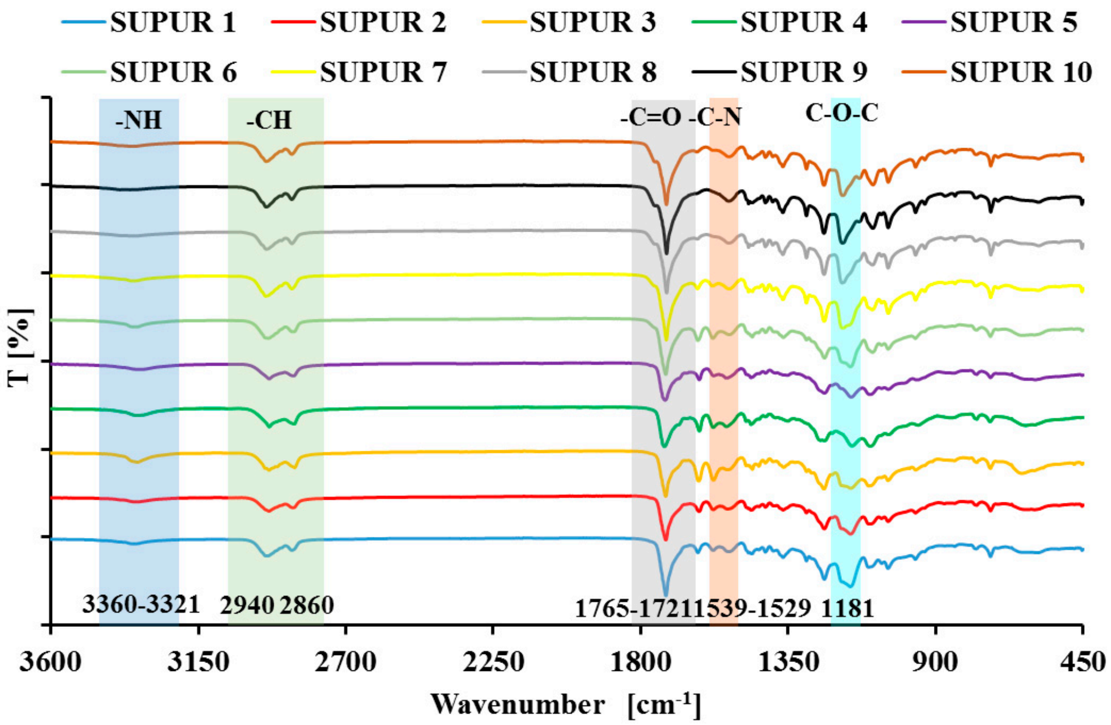

Figure S4. Stacked IR spectra of SUPURs 1 - 10.

Table S1. The masses m_1 and m_2 are the initial weight, and swollen weight in water for the SUPUR polymers, respectively.

Sample name	m_1 (g)	m_2 (g)	Absorbed water (%)
SUPUR 1	0.087	0.089	2.2
SUPUR 2	0.100	0.104	4.0
SUPUR 3	0.116	0.123	6.0
SUPUR 4	0.056	0.069	23.3
SUPUR 5	0.0770	0.104	35.5
SUPUR 6	0.084	0.085	1.3
SUPUR 7	0.107	0.133	24.3
SUPUR 8	0.098	0.121	23.2
SUPUR 9	0.100	0.123	12.0
SUPUR 10	0.1144	0.148	29.3

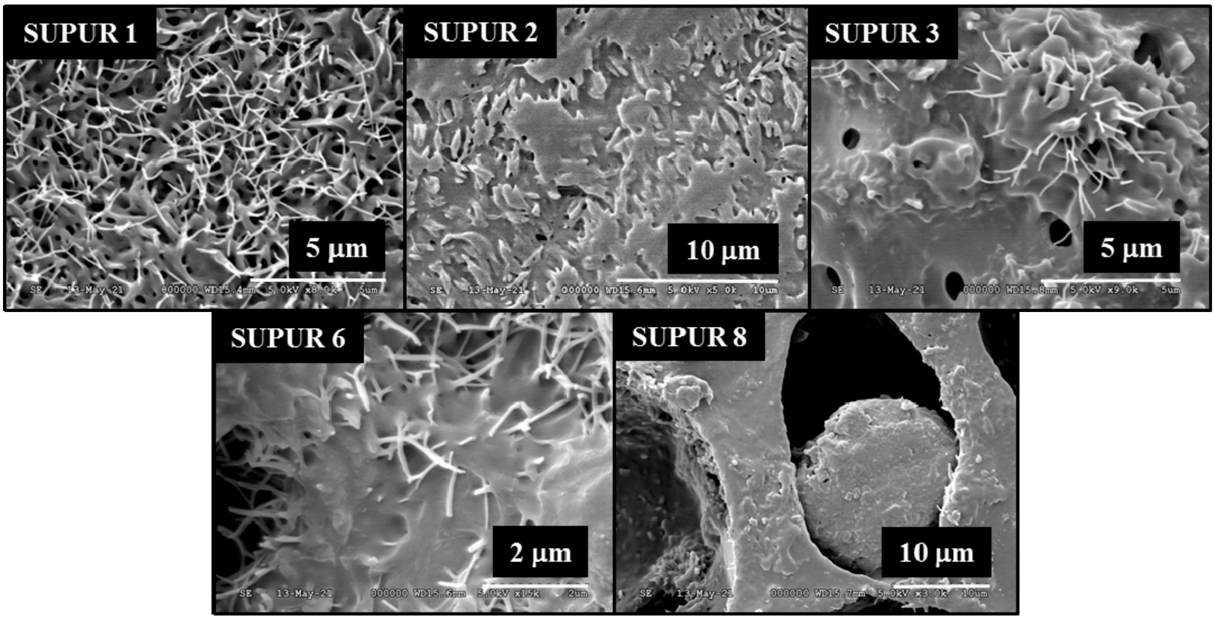

Figure S5. Magnified SEM images of SUPURs 1 - 3 and 6, 8.

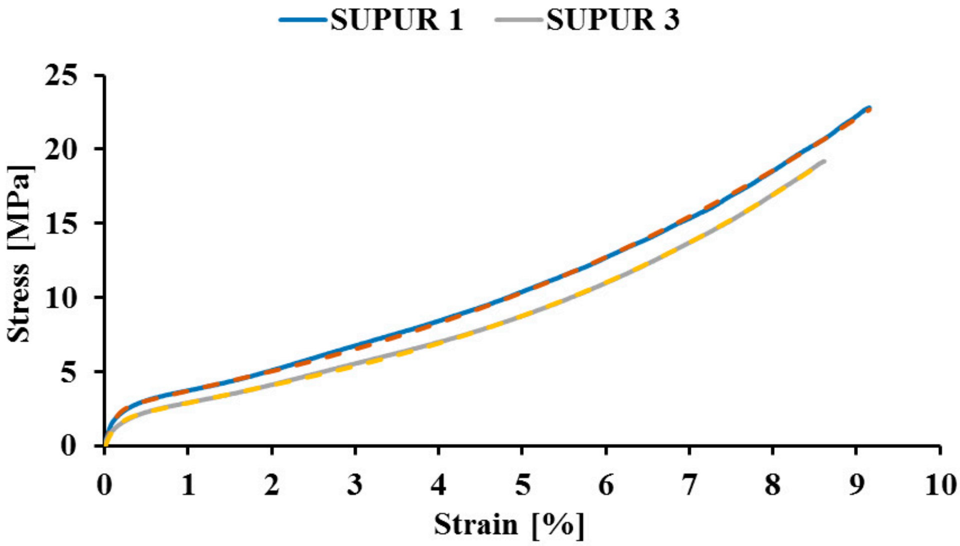

Figure S6. The stress-strain curves of SUPURs 1 and 3. The solid lines reperesented the experimental data, while dashed lines stand for fitted curves.

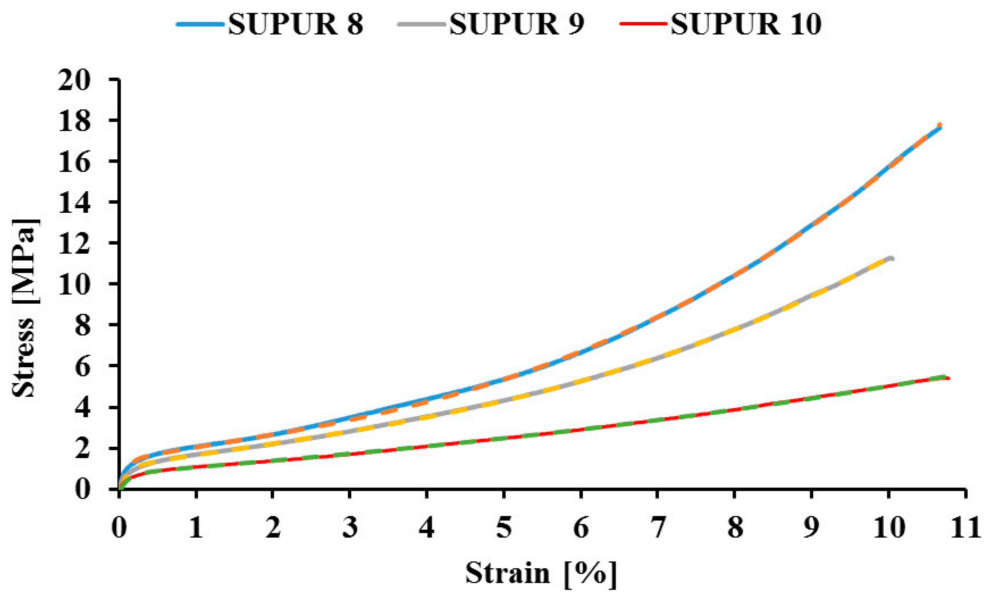

Figure S7. The stress-strain curves of SUPURs 8 - 10. The solid lines reperesented the experimental data, while dashed lines stand for fitted curves.

Table S2. The fitted parameters of Equations 5 - 7 for samples SUPUR 1 - 4 and 6 - 10.

	$[(d\varepsilon/dt)a_1]^{-1}$	a_2 (MPa)	$a_3(d\varepsilon/dt)$ (MPa)	α	β
SUPUR 1	10.03	1.32	2.52	0.07	1.34
SUPUR 2	9.27	1.52	2.81	0.05	1.56
SUPUR 3	8.15	1.20	1.84	0.04	1.64
SUPUR 4	8.55	1.27	1.85	0.05	1.24
SUPUR 6	8.82	1.42	2.46	0.07	1.70
SUPUR 7	8.52	0.67	1.13	0.05	1.45
SUPUR 8	10.96	0.63	1.46	0.03	1.60
SUPUR 9	9.55	0.57	1.14	0.01	1.76
SUPUR 10	8.50	0.33	0.77	0.00	1.49

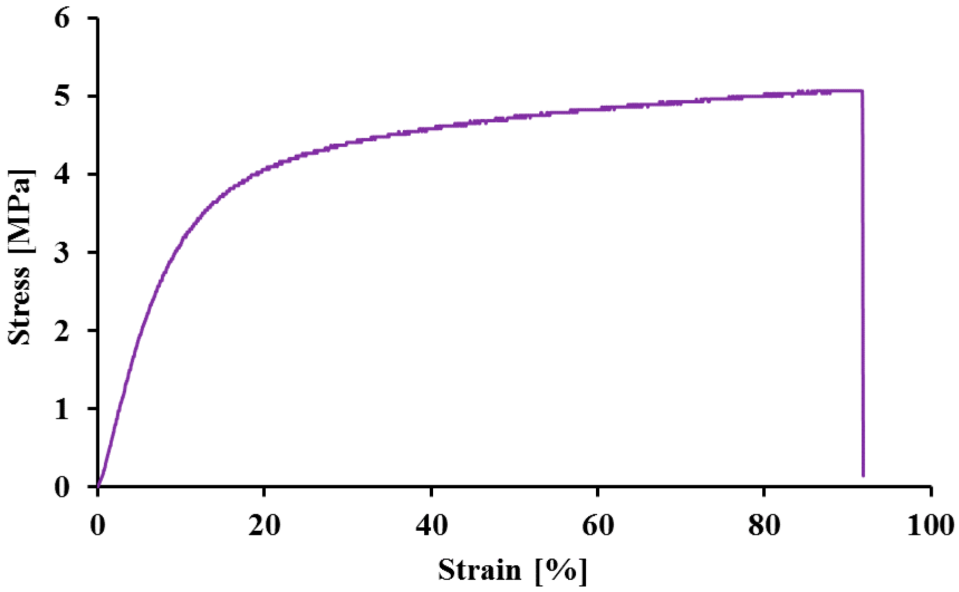

Figure S8. The stress-strain curves of SUPUR 5.

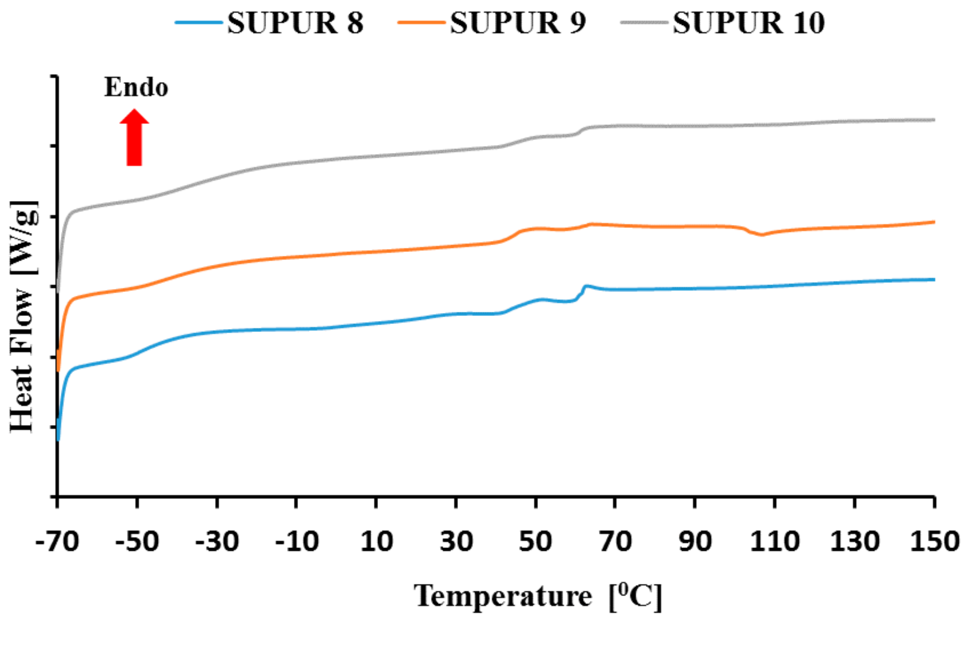

Figure S9. DSC curves of SUPUR 8 - 10.

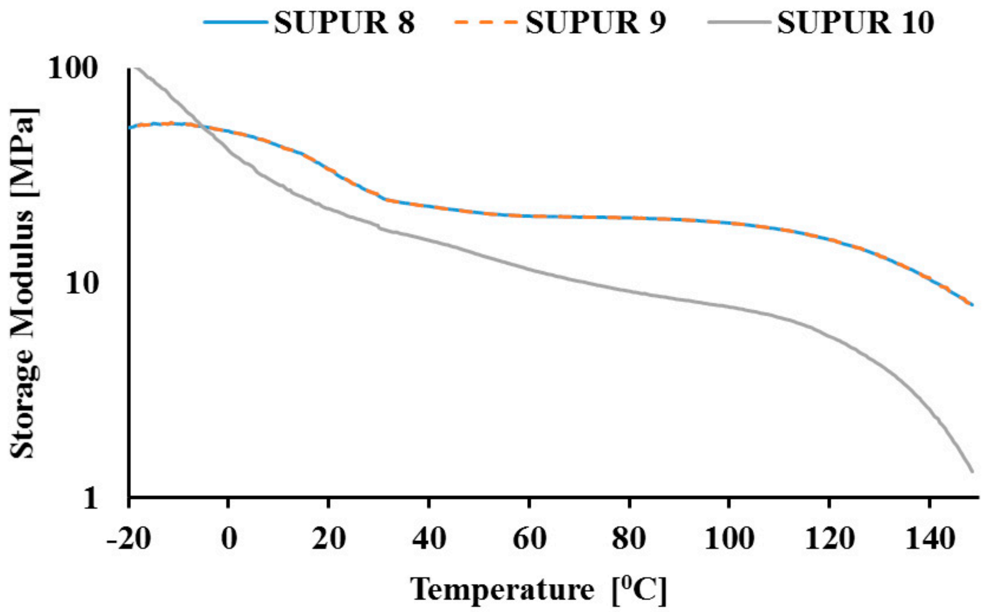

Figure S10. Storage modulus curves of SUPUR 8 - 10.

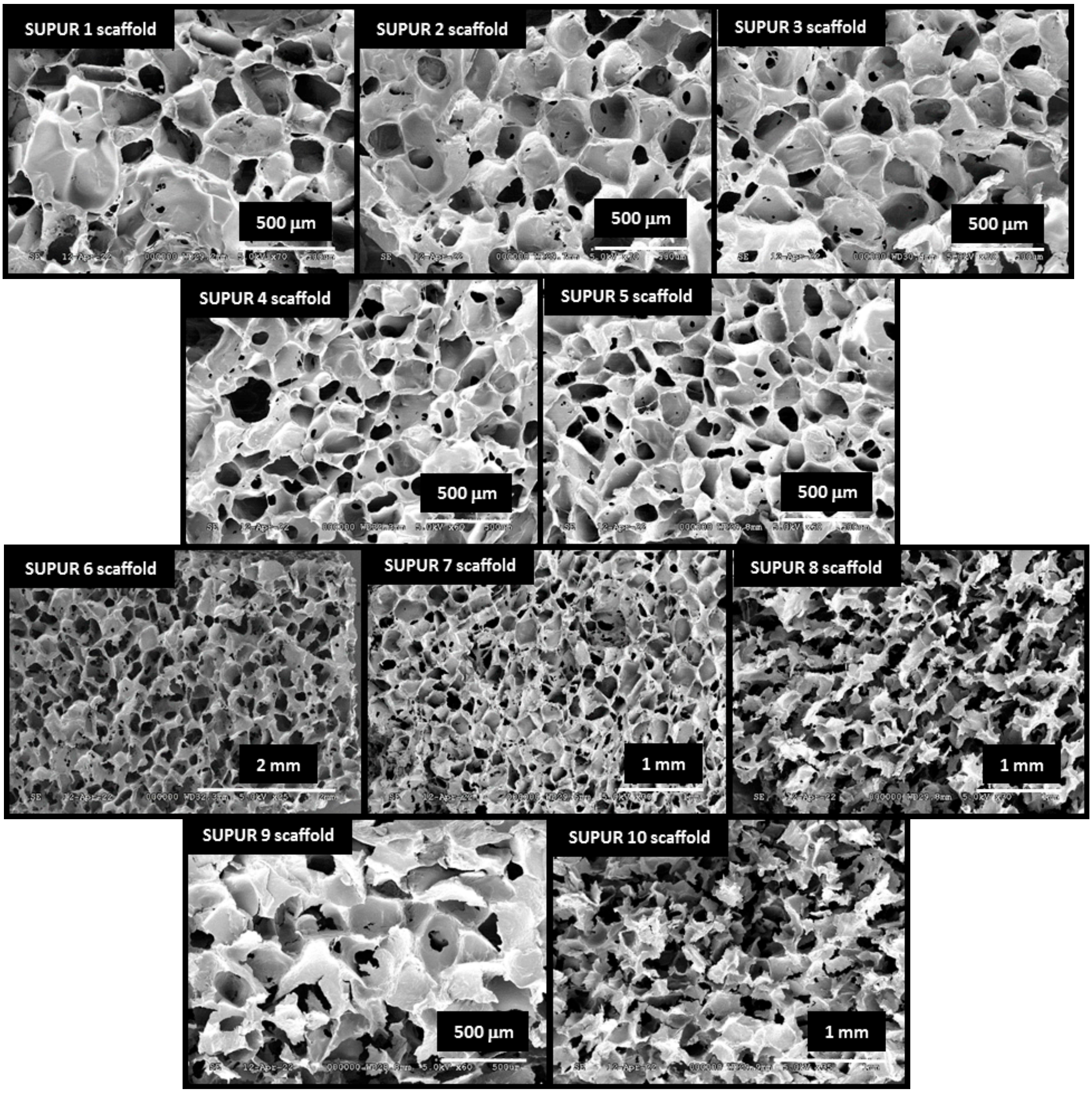

Figure S11. SEM images of SUPUR 1 - 10 scaffolds.

Details about the dental pulp stem cells used in this study

"Human dental pulp stem cells (DPSCs) were isolated from the pulp tissue of healthy human wisdom teeth as it was described previously [1], and were sorted for STRO-1 cell surface marker [2] (patient declaration of agreement No. F0102/1ST).

For our work, we used impacted winsdom teeth surgically removed from patients at the University of Debrecen Faculty of Dentistry, taking care to select teeth that were not in contact with the oral cavity and that were not damaged during removal.

The wisdom teeth were placed in sterile Hank's saline solution immediately after surgical process. The winsdom teeth were separated longitudinally with a turbinate diamond fissure drill under water cooling, taking care to ensure sterility and the integrity of the pulp chamber and root canal, and immediately placed in sterile Hank's saline supplemented with streptomycin penicillin at room temperature and transported to the laboratory. In a laminar chamber, teeth were split in half along the incision under sterile conditions and pulp was removed. Pulp was ground with a sterile scalpel and digested for one hour at 37°C with 1.5 ml per sample of 3 mg/ml collagenase (Sigma-Aldrich, St. Louis, MO, USA) and 4 mg/ml dyspase (Gibco, Life Technologies, Grand Island, NY, USA) in Hank's saline solution. Samples were vortexed every 15 to 20 min during digestion and then centrifuged at 1200 rpm for 5 min. The supernatant was removed, the cell culture was supplemented with 10 % fetal bovine serum (FBS), 1 % penicillin-strep tomycin (Sigma-Aldrich, St. Louis, MO, USA) and 1% GlutaMAX (Gibco, Life Technologies, Grand Island, NY, USA) Minimum Essential Medium Eagle Alpha modification (Sigma-Aldrich, St. Louis, MO, USA) were suspended in culture medium and plated in 25 cm^2 cell culture flasks. The isolated heterogeneous cell populations were cultured at 37 °C under 100 % humidity and 5 % CO_2. The cell culture medium was changed three times a week.

Fluorescence sorting was performed using a BD FACS Aria III flow cytometer (BD Biosciences, San Jose, CA, USA). Selected STRO-1 positive cells were cultured in the medium and under the conditions previously mentioned."

1. Kadar K, Kiraly M, Porcsalmy B, Molnar B, Racz GZ, Blazsek J, et al. Differentiation potential of stem cells from human dental origin—promise for tissue engineering. J Physiol Pharmacol. 2009;60(Suppl 7):167–75.
2. Kerenyi F, Tarapcsak S, Hrubi E, Barathne SA, Hegedus V, Balogh S, et al. Comparison of sorting of fluorescently and magnetically labeled dental pulp stem cells. Fogorv Sz. 2016;109(1):29–33
